# Pathological fractures in pediatric extremities: a comparative analysis of patient characteristics, trauma mechanisms, and lesion types

**DOI:** 10.1007/s00431-026-07181-1

**Published:** 2026-06-26

**Authors:** Clemens Clar, Jürgen Rünk, Patrick Reinbacher, Vanessa Sackl, Andreas Leithner, Tanja Kraus

**Affiliations:** 1 Paediatric Orthopaedic Unit, Department of Orthopaedics and Trauma, Medical University of Graz, Graz, Austria; 2 Department of Paediatric Surgery, Tartu University Hospital, Tartu, Estonia; 3 Department of Orthopaedics and Trauma, Medical University of Graz, Graz, Austria

**Keywords:** Pathological fracture, Pediatric fracture, Pediatric orthopaedics, Bone lesions, Fracture epidemiology

## Abstract

Pathological fractures in children are uncommon manifestations of underlying bone lesions. Differences in biomechanical loading and lesion distribution between the upper and lower extremities may result in distinct fracture characteristics and clinical presentations. The aim of this study was to characterize pathological fractures in the growing skeleton and to identify location-specific differences between upper- and lower-extremity fractures regarding patient demographics, trauma mechanisms, fracture characteristics, and underlying lesion types. This retrospective single-center study included 235 pediatric patients (0–17 years) with radiologically confirmed pathological fractures treated between 1999 and 2021. Demographic, clinical, and radiographic data were extracted from institutional records. Fractures were classified according to trauma mechanism, accident setting, anatomical location and underlying pathology. Statistical comparisons between upper- and lower-extremity fractures were performed using standard statistical tests, with statistical significance defined as *p* < 0.05. A total of 235 pathological fractures were analyzed, including 120 fractures of the upper extremity (51.1%) and 115 fractures of the lower extremity (48.9%). Age distribution was comparable between groups (median age 11 years; *p* = 0.887). Female patients were significantly more likely to sustain a pathological fracture of the lower extremity than of the upper extremity (38.3% vs. 25.0%; OR 1.86, 95% CI 1.06–3.25; *p* = 0.041). Significant differences were observed regarding accident setting and trauma mechanism (*p* = 0.011 and *p* = 0.0015, respectively). Lower-extremity fractures occurred more frequently in the absence of trauma or following low-energy trauma, whereas upper-extremity fractures were predominantly associated with falls on level surfaces. Malignant lesions were more common among lower-extremity fractures than upper-extremity fractures (9.6% vs. 3.3, *p* = 0.063).

*Conclusion:* Pathological fractures of the upper and lower extremities in children demonstrate distinct clinical characteristics. Lower-extremity fractures were more frequently associated with absent or low-energy trauma and showed a trend toward a higher proportion of malignant underlying lesions, whereas upper-extremity fractures predominantly resulted from falls on level surfaces. These location-specific differences suggest that fracture location may provide valuable diagnostic information and should be considered during the evaluation of children presenting with pathological fractures.

**Table:** 

**What is Known:** • *Pathological fractures in children may be the first manifestation of underlying skeletal pathology.* • *Fractures after minimal trauma warrant careful diagnostic evaluation.*
**What is New:** • *Lower-extremity pathological fractures were more frequently associated with absent or low-energy trauma and a higher burden of systemic or multifocal bone disorders.* •* Lower-extremity fractures showed a trend toward a higher prevalence of malignant underlying lesions, supporting their value as a diagnostic red flag.*

**What is Known:**

• *Pathological fractures in children may be the first manifestation of underlying skeletal pathology.*

• *Fractures after minimal trauma warrant careful diagnostic evaluation.*

**What is New:**

• *Lower-extremity pathological fractures were more frequently associated with absent or low-energy trauma and a higher burden of systemic or multifocal bone disorders.*

•* Lower-extremity fractures showed a trend toward a higher prevalence of malignant underlying lesions, supporting their value as a diagnostic red flag.*

## Introduction

Pathological fractures in children and adolescents represent a rare but clinically important subset of pediatric skeletal injuries [1–3]. In contrast to traumatic fractures occurring in otherwise healthy bone, pathological fractures arise in bone weakened by an underlying local or systemic condition, such as benign or malignant bone tumors, infection, metabolic bone disease, or genetic disorders affecting bone integrity. Importantly, a pathological fracture may be the first clinical manifestation of an underlying disorder, making early recognition essential for appropriate diagnostic evaluation and management [3–5].

Although the epidemiology and etiology of fractures in the growing skeleton in general have been studied [1, 3] data specifically addressing pathological fractures according to anatomical location in the growing skeleton remain limited. Distinguishing pathological fractures from traumatic injuries can be challenging in clinical practice, particularly when fractures occur after low-energy or seemingly adequate trauma. Failure to recognize an underlying pathology may delay diagnosis and appropriate treatment [6–9].

The anatomical location of a pathological fracture may provide important diagnostic information. The upper and lower extremities differ substantially with regard to biomechanical loading, functional demands and growth dynamics [8, 10]. These factors may influence fracture occurrence, injury mechanisms and clinical presentation. However, location-specific characteristics of pediatric pathological fractures have received little attention, and differences between upper- and lower-extremity fractures remain poorly defined. A better understanding of such patterns may facilitate recognition of the underlying pathology and support clinical decision-making in children presenting with pathological fractures [11, 12].

Therefore, the aim of this study was to characterize pathological fractures in the growing skeleton and to identify location-specific differences between upper- and lower-extremity fractures regarding patient demographics, trauma mechanisms, fracture characteristics, and underlying lesion types.

## Materials and methods

### Study design and ethics approval

This retrospective single-center observational study analyzed patients with pathological fractures treated at a Level I pediatric trauma center between September 1999 and December 2021. Children and adolescents aged 0 to 17 years at the time of injury were eligible for inclusion. All patients were skeletally immature, with open growth plates at the time of injury.

This study was approved by the Ethics Committee of the Medical University of Graz (EK: 34–350 ex 21_22 1154–202). Informed consent was waived as per the retrospective nature of the study. The study was performed in accordance with the Good Clinical Practice (GCP) guideline and the Declaration of Helsinki. All data were anonymized prior to analysis in accordance with institutional and legal data protection regulatory.

### Diagnostic assessment

Classification was based on radiological evidence of an underlying bone lesion considered causative for the fracture. The initial diagnosis was based on the X-ray, when clinically indicated, diagnostic confirmation was supported by advanced imaging modalities, including computed tomography (CT) or magnetic resonance imaging (MRI), and/or histopathological examination.

Initial fracture diagnosis was established using plain radiographs and additional CT depending on clinical indication. All images were reviewed and confirmed by a board-certified radiologist. MRI was performed selectively to further characterize lesion morphology, assess soft tissue involvement, or clarify equivocal findings. Imaging of the entire affected bone was routinely performed to assess lesion extent. To characterize lesion location more precisely in relation to the physis, lesions were subclassified as epiphyseal, metaphyseal, diaphyseal, or combined.

### Data collection and variables

Clinical data were retrospectively extracted from the institutional electronic health information system (MEDocs). Data extraction was independently performed by four investigators (J.R. P.R., V.S., and C.C.) to minimize extraction bias, with discrepancies resolved by consensus. Collected variables included demographic data (age at injury and sex), injury-related characteristics (date, mechanism, and setting of injury), and fracture-related parameters (affected bone, laterality, anatomical location, fracture morphology, and joint involvement). A fall on a level surface was defined as a fall from standing height without vertical displacement (e.g., walking, running, or sports-related falls). A fall from height < 3 m was defined as any fall involving a vertical drop of less than 3 m above ground level. Treatment-related variables included the initial management strategy for fracture stabilization (operative versus conservative therapy). Specific treatment modalities were categorized as non-operative management, internal fixation without documented biopsy or curettage, biopsy with internal fixation, curettage/grafting/defect filling with or without fixation, oncologic resection/reconstruction/amputation, and other operative treatment. Biopsy status and revision surgery were recorded when available.

### Age groups and outcomes

To account for developmental differences in skeletal maturity, patients were stratified into predefined age groups reflecting key stages of growth: infants up to 1 year of age, pre-school children aged > 1–6 years, pre-pubertal school-aged children aged > 6–10 years, early adolescents aged > 10–14 years, and late adolescents aged > 14–17 years. Patients were grouped according to fracture location (upper vs. lower extremity) for comparative analysis. Primary outcomes included location-specific differences between upper- and lower-extremity pathological fractures regarding patient demographics, trauma characteristics, fracture characteristics, and underlying pathology. Secondary outcomes included treatment modality and age-group distribution.

### Statistical analysis

The dataset was compiled using Excel® (Microsoft® Corporation, Redmond, WA, USA) for statistical evaluation. Statistical analysis was performed using descriptive methods. Continuous variables were summarized as means with standard deviations or medians with ranges, as appropriate. Categorical variables were reported as absolute numbers and percentages. Comparisons between upper- and lower-extremity pathological fracture groups were conducted using chi-square or Fisher’s exact tests for categorical variables and Mann–Whitney U test for continuous variables, depending on data distribution.

Effect sizes for categorical comparisons were calculated using Cramér’s V to quantify the strength of associations. All tests were two-sided, and a *p* value < 0.05 was considered statistically significant. Owing to the limited number of pathological fractures, multivariable analysis was not performed. Univariable logistic regression analyses were performed to identify factors associated with operative treatment.

## Results

A total of 235 pathological fractures were included in the analysis. Of these, 120 fractures (51.1%) involved the upper extremity and 115 fractures (48.9%) the lower extremity. The overall cohort comprised significantly more male patients (*n* = 162, 68.9%) than female patients (*n* = 73, 31.1%) (*p* < 0.001) (Table 1).

**Table 1:** Comparison of patient characteristics, trauma-related factors, fracture features, treatment strategies, and underlying lesion type between upper- and lower-extremity pathological fractures

Variable	Upper extremity (*n* = 120)	Lower extremity (*n* = 115)	Test	*p*-value	Effect size/OR	95% CI
Age, years	10.6 ± 3.7; median 11	10.3 ± 4.7; median 11	Mann–Whitney U	0.697	–	–
Female sex	30 (25.0%)	43 (37.4%)	Chi-square	0.040	OR 1.79	1.02–3.14
Accident site (overall)	–	–	Chi-square	0.013	Cramér’s V 0.27	–
No adequate trauma	6 (5.9%)	21 (21.2%)	Fisher`s exact	0.0017	OR 4.31	1.66–11.20
Educational institution	21 (20.6%)	11 (11.1%)		–	–	–
Accident mechanism (overall)	–	–	Chi-square	0.0015	Cramér’s V 0.29	–
Other/atypical mechanism	23 (19.2%)	44 (38.3%)		–	–	–
Side (left)	59/118 (50.0%)	58/114 (50.9%)	Chi-square	0.945	Cramér’s V 0.01	–
Fracture location across anatomical regions	Epiphyseal:6 (5.0%) Metaphyseal: 44 (36.7%) Combined: 8 (7.5%) Diaphyseal:62 (51.7%)	Epiphyseal: 4 (3.5%) Metaphyseal: 49 (42.6%) Combined: 9 (7.8%) Diaphyseal:53 (46.1%)	Chi-square	0.758	Cramér’s V 0.09	–
Joint involvement	3 (2.5%)	3 (2.6%)	Fisher`s exact	1.0	–	–
Operative treatment	70 (58.3%)	68 (59.1%)	Chi-square	0.901	Cramér’s V 0.01	–
Malignant lesion	4 (3.3%)	11 (9.6%)	Fisher's exact	0.063	OR 3.07	0.95–9.93

### Demographic characteristics

Age did not differ significantly between fracture locations. In the upper-extremity group, the mean age was 10.6 ± 3.7 years (median 11 years, interquartile range [IQR] 8.0–13.0), compared with 10.3 ± 4.7 years (median 11 years, IQR 7.0–14.0) in the lower-extremity group (*p* = 0.697).

Sex distribution differed significantly between groups. Female patients accounted for a greater proportion of lower-extremity fractures than upper-extremity fractures (43/115, 37.4% vs. 30/120, 25.0%; odds ratio [OR] 1.79, 95% CI 1.02–3.14; *p* = 0.040). Conversely, male patients were more frequently affected by upper-extremity fractures (90/120, 75.0% vs. 72/115, 62.6%; OR 0.56, 95% CI 0.32–0.98).

### Trauma characteristics

**Fig. 1:**
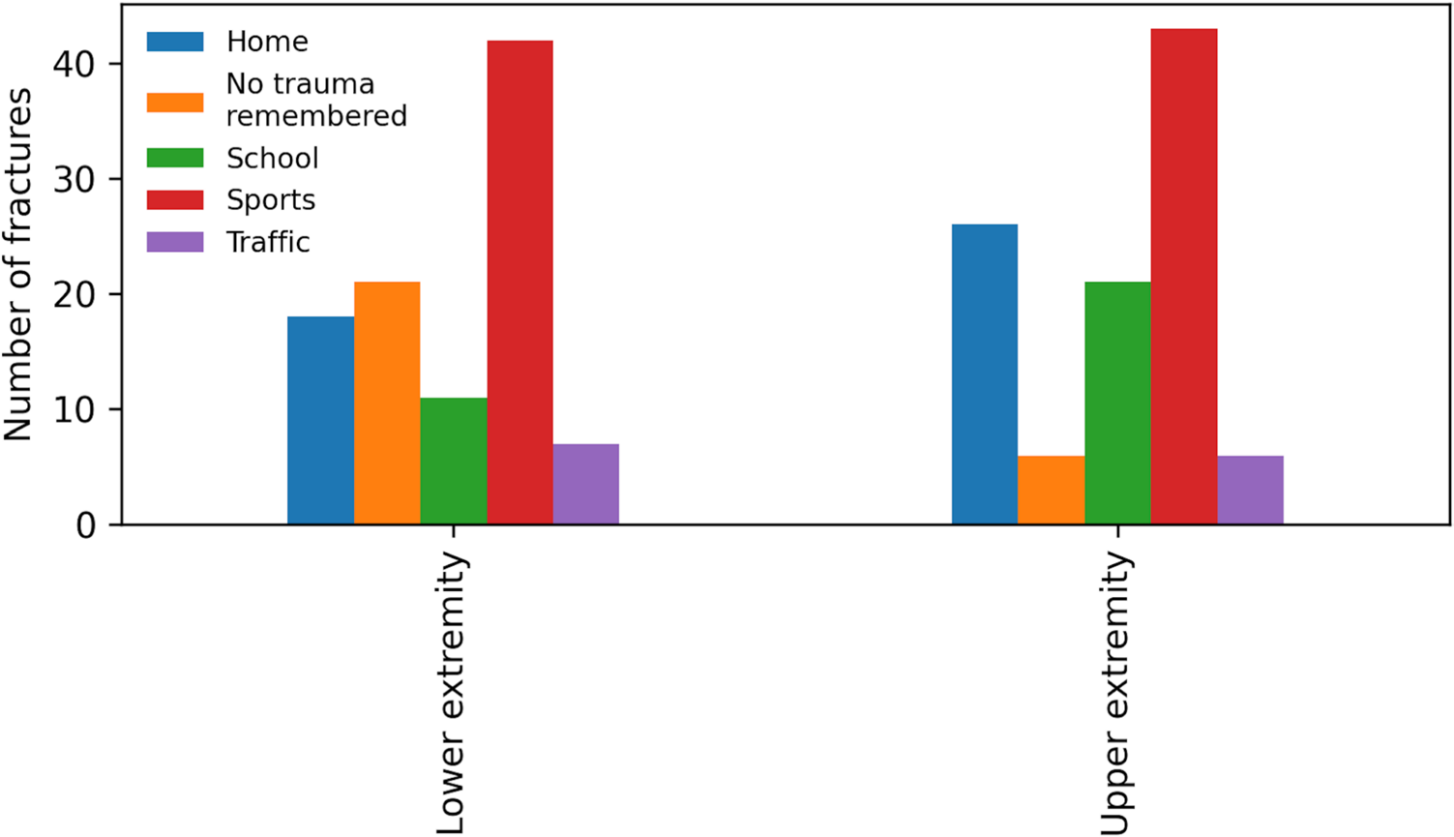
Accident site of pathological fractures stratified by anatomical location

Accident setting differed significantly between upper- and lower-extremity fractures (*p* = 0.013, Cramér’s V = 0.27). Among cases with documented accident setting (*n* = 201), fractures without a recalled or adequate traumatic event were substantially more common in the lower extremity than in the upper extremity (21/99, 21.2% vs. 6/102, 5.9%). In contrast, injuries occurring in educational institutions were more frequent in the upper extremity (21/102, 20.6%) than in the lower extremity (11/99, 11.1%). Fractures sustained at sports or leisure facilities occurred at similar proportions in both groups (43/102, 42.2% vs. 42/99, 42.4%), while home-related injuries were somewhat more frequent in the upper extremity (26/102, 25.5% vs. 17/99, 17.2%). One additional lower-extremity fracture was classified as other accident setting (1/99, 1.0%) (Fig. 1).

**Fig. 2:**
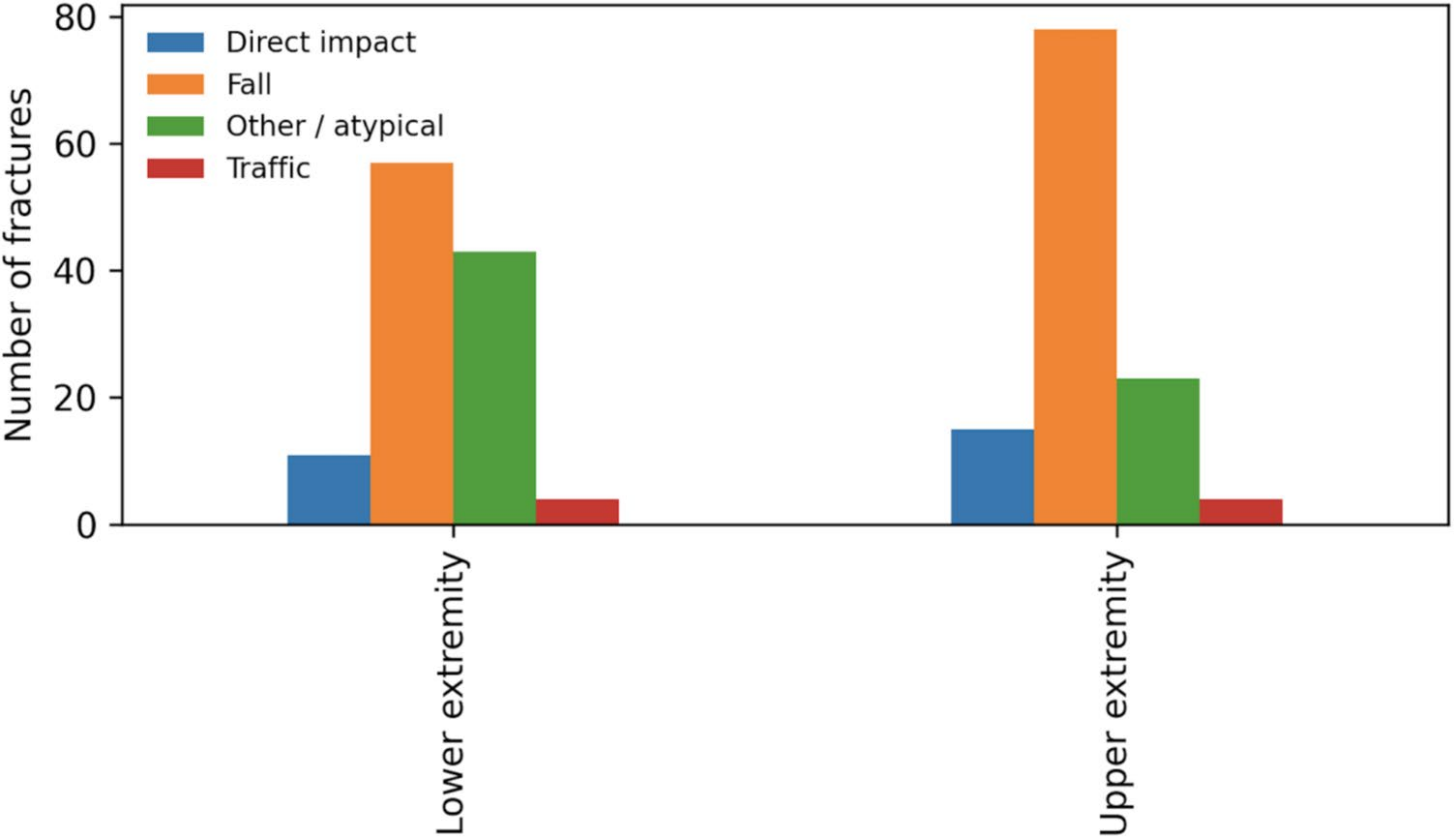
Trauma mechanism of pathological fractures stratified by anatomical location

Trauma mechanism also differed significantly between groups (*p* = 0.0015, Cramér’s V = 0.29). Upper-extremity fractures most resulted from falls on level surfaces (71/120, 59.2%), whereas this mechanism was less frequent in lower-extremity fractures (40/115, 34.8%). By contrast, lower-extremity fractures were more often associated with mechanisms categorized as “other” (44/115, 38.3% vs. 23/120, 19.2%). Falls from a height < 3 m were also more common in the lower extremity (12/115, 10.4%) than in the upper extremity (6/120, 5.0%), whereas crush or direct-impact injuries occurred at comparable rates (11/115, 9.6% vs. 15/120, 12.5%). Traffic-related mechanisms were uncommon in both groups (4/115, 3.5% vs. 4/120, 3.3%). Falls from a height > 3 m were rare but occurred more often in lower-extremity than upper-extremity fractures (4/115, 3.5% vs. 1/120, 0.8%) (Fig. 2).

### Fracture characteristics

No significant differences were observed between upper- and lower-extremity fractures regarding laterality or joint involvement. Fractures were almost evenly distributed between the left and right side among classifiable cases (upper extremity: 59 left, 59 right, two bilateral; lower extremity: 58 left, 57 right *p* = 0.945).

Likewise, the distribution of fracture location across anatomical regions did not differ significantly between groups (*p* = 0.758). Diaphyseal fractures represented the most frequent pattern in both the upper extremity (62/120, 51.7%) and lower extremity (53/115, 46.1%), followed by metaphyseal fractures (44/120, 36.7% and 49/115, 42.6%, respectively). Epiphyseal involvement was uncommon (6/120, 5.0% vs. 4/115, 3.5%). Combined metaphyseal-diaphyseal fractures were observed in 5/120 upper-extremity fractures (4.2%) and 4/115 lower-extremity fractures (3.5%), while combined epiphyseal-metaphyseal involvement was present in 3/120 (2.5%) and 5/115 (4.3%), respectively. Joint involvement was infrequent overall and observed in only 3 cases in each group (2.5% vs. 2.6%; *p* = 1.0).

### Treatment

General treatment strategy did not differ by fracture location. Operative management was performed in 70/120 upper-extremity fractures (58.3%) and 68/115 lower-extremity fractures (59.1%), whereas conservative treatment was used in 50/120 (41.7%) and 47/115 (40.9%), respectively (*p* = 0.901).

The distribution of specific treatment modalities among the 235 fractures differed significantly between upper- and lower-extremity fractures (*p* = 0.001, Cramér’s V = 0.30). Internal fixation without documented biopsy or curettage was more frequently performed in lower-extremity fractures (28/115, 24.3%) than in upper-extremity fractures (8/120, 6.7%). In contrast, biopsy combined with internal fixation was more common in upper-extremity fractures (35/120, 29.2%) than in lower-extremity fractures (15/115, 13.0%). Curettage, grafting, or defect filling with or without fixation was performed in 48/235 patients (20.4%), with comparable proportions in upper- and lower-extremity fractures (26/120, 21.7% vs. 22/115, 19.1%). Oncologic resection, reconstruction, or amputation was required in 3/235 patients (1.3%). One lower-extremity fracture associated with osteomyelitis was classified as other operative treatment (1/235, 0.4%).

Age distribution did not differ significantly across specific treatment modalities (*p* = 0.592). Median age was comparable between non-operative management (11.0 years, IQR 7.0–14.0), internal fixation without documented biopsy or curettage (11.0 years, IQR 7.8–14.3), biopsy with internal fixation (10.5 years, IQR 8.0–13.0), and curettage, grafting, or defect filling with or without fixation (11.0 years, IQR 9.0–13.0).

Biopsy status was available in 203 patients. Overall, 165/203 patients (81.3%) underwent biopsy, without a significant difference between upper- and lower-extremity fractures (97/116, 83.6% vs. 68/87, 78.2%; *p* = 0.324). Revision data were available in 220 patients. Revision surgery was documented in 62/220 cases (28.2%) and did not differ significantly between upper- and lower-extremity fractures (34/111, 30.6% vs. 28/109, 25.7%; *p* = 0.415).

In univariable analysis, operative treatment was not associated with lower-extremity location (OR 1.03, 95% CI 0.61–1.74; *p* = 0.901), female sex (OR 1.01, 95% CI 0.58–1.77; *p* = 0.970), malignancy (OR 1.06, 95% CI 0.36–3.08; *p* = 0.917), or absent/non-adequate trauma (OR 0.75, 95% CI 0.42–1.33; *p* = 0.326). Diaphyseal fracture location was associated with higher odds of operative treatment (OR 2.44, 95% CI 1.43–4.17; *p* < 0.001), whereas metaphyseal fractures were associated with lower odds of operative treatment (OR 0.53, 95% CI 0.31–0.91; *p* = 0.020).

### Underlying lesion type

Malignant lesions were more frequently observed in lower-extremity fractures than in upper-extremity fractures (11/115, 9.6% vs. 4/120, 3.3%), without statistical significance (*p* = 0.063), although the estimated odds ratio suggested a more than threefold increase in the odds of malignancy for lower-extremity fractures (OR 3.07, 95% CI 0.95–9.93). Accordingly, benign or non-malignant lesions accounted for 104/115 lower-extremity fractures (90.4%) and 116/120 upper-extremity fractures (96.7%).

The distribution of underlying entities differed substantially between upper- and lower-extremity fractures (*p* < 0.001, Cramér’s V = 0.64). Bone cysts were markedly more common in the upper extremity (90/120, 75.0%) than in the lower extremity (28/115, 24.3.%).

The most common underlying lesions overall were bone cysts (118/235, 50.2%), non-ossifying fibroma (29/235, 12.3%), osteoporosis (19/235, 8.1%), enchondroma (14/235, 6.0%), osteosarcoma (9/235, 3.8%), fibrous dysplasia (8/235, 3.4%), osteomyelitis (7/235, 3.0%), and osteogenesis imperfecta (7/235, 3.0%). Malignant lesions accounted for 15/235 cases (6.4%) and included osteosarcoma, chondrosarcoma, Ewing sarcoma, neuroblastoma, and hemangiopericytoma. Patients with malignant lesions were significantly older than those with benign or non-malignant lesions (median 14 years, IQR 10–15 vs. 11 years, IQR 7–13; *p* = 0.013) (Table 2).

**Table 2:** Distribution of underlying pathological entities in pediatric extremity fractures

Underlying entity	Entity group	Overall (*N* = 235)	Upper extremity (*n* = 120)	Lower extremity (*n* = 115)
Bone cyst	Benign/localized	119 (50.6)	90 (75.0)	29 (25.2)
Non-ossifying fibroma	Benign/localized	29 (12.3)	5 (4.2)	24 (20.9)
Osteoporosis	Systemic/multifocal	19 (8.1)	2 (1.7)	17 (14.8)
Enchondroma	Benign/localized	14 (6.0)	12 (10.0)	2 (1.7)
Osteosarcoma	Malignant	9 (3.8)	2 (1.7)	7 (6.1)
Fibrous dysplasia	Benign/localized	8 (3.4)	1 (0.8)	7 (6.1)
Osteogenesis imperfecta	Systemic/multifocal	7 (3.0)	0 (0.0)	7 (6.1)
Osteomyelitis	Infectious	7 (3.0)	3 (2.5)	4 (3.5)
Stress fracture	Other/non-neoplastic	5 (2.1)	0 (0.0)	5 (4.3)
Non-ossifying fibroma + bone cyst	Benign/localized	4 (1.7)	2 (1.7)	2 (1.7)
Chondrosarcoma	Malignant	3 (1.3)	2 (1.7)	1 (0.9)
Osteopenia	Systemic/multifocal	3 (1.3)	0 (0.0)	3 (2.6)
Chondroblastoma	Benign/localized	1 (0.4)	0 (0.0)	1 (0.9)
Eosinophilic granuloma	Benign/localized	1 (0.4)	0 (0.0)	1 (0.9)
Epiphyseal dysplasia	Systemic/multifocal	1 (0.4)	0 (0.0)	1 (0.9)
Ewing sarcoma	Malignant	1 (0.4)	0 (0.0)	1 (0.9)
Hemangiopericytoma	Malignant	1 (0.4)	0 (0.0)	1 (0.9)
Muscular dystrophy	Systemic/multifocal	1 (0.4)	0 (0.0)	1 (0.9)
Myofibroma	Benign/localized	1 (0.4)	1 (0.8)	0 (0.0)
Neuroblastoma	Malignant	1 (0.4)	0 (0.0)	1 (0.9)

When benign or non-malignant lesions were further stratified, 7/220 cases (3.2%) were infectious and 213/220 cases (96.8%) were non-infectious. Not all fractures occurred in solitary lesions. Overall, 31/235 patients (13.2%) had systemic or multifocal bone conditions, including osteoporosis, osteopenia, osteogenesis imperfecta, muscular dystrophy, or epiphyseal dysplasia. These conditions were significantly more common in lower-extremity fractures than in upper-extremity fractures (29/115, 25.2% vs. 2/120, 1.7%; *p* < 0.001, Cramér’s V = 0.35).

## Discussion

This study identified location-specific differences between pathological fractures of the upper and lower extremities. Lower-extremity fractures were more frequently associated with absent or low-energy trauma and showed a higher proportion of malignant lesions compared with upper-extremity fractures. Furthermore, sex distribution differed between fracture locations.

Upper-extremity pathological fractures were more common in male patients, whereas lower-extremity fractures occurred more frequently in female patients. Sex-related differences in pediatric fracture epidemiology have been reported previously and are often attributed to variations in activity patterns, skeletal maturation, and hormonal influences during growth [7, 13]. In the context of pathological fractures, however, sex should be regarded as a contextual rather than a determinative variable. The observed difference may reflect differences in exposure, activity patterns, skeletal maturation, or the distribution of underlying lesions, but it should not independently guide imaging or treatment decisions. In clinical practice, sex may contribute to the overall pre-test probability of certain conditions, but trauma adequacy, radiographic lesion morphology, and symptoms preceding the fracture remain substantially more important. Data specifically examining sex differences in pathological fractures are limited [8, 14]. These findings suggest that sex may influence fracture location in the presence of underlying bone pathology, although the underlying mechanisms remain unclear.

Trauma-related characteristics differed between fracture locations. Lower-extremity pathological fractures were more frequently associated with absent or low-energy trauma, whereas upper-extremity fractures most resulted from falls on level surfaces. The higher proportion of trauma-associated upper-extremity fractures may be explained by protective reflexes during falls, leading to direct load transmission to the arms, as well as greater exposure of the upper extremities during play and sports activities. In contrast, the weight-bearing nature of the lower extremities may predispose to fractures in weakened bone even in the absence of significant trauma. These findings are clinically relevant, as fractures without a clear traumatic mechanism should raise suspicion of an underlying pathological condition. In particular, atraumatic or minimally traumatic fractures of the lower extremity may represent an important diagnostic red flag and warrant thorough radiological evaluation of the entire affected bone [14–17].

The observed differences may reflect the distinct biomechanical demands of the upper and lower extremities. Whereas upper-extremity fractures frequently occur following direct load transmission during falls, the weight-bearing function of the lower extremity may predispose weakened bone to fracture even in the absence of substantial trauma. This concept is further supported by the higher proportion of systemic or multifocal bone disorders and the trend toward a greater proportion of malignant lesions observed in lower-extremity fractures.

In contrast, fracture-related characteristics such as laterality, fracture location, and treatment strategy did not differ significantly between upper- and lower-extremity fractures. Joint involvement was rare, consistent with recent literature, suggesting that metaphyseal and physeal regions are the most common locations of underlying lesions in children [17–19]. Operative management was required in approximately 60% of cases in both groups, reflecting the need to address both fracture stability and the underlying pathology.

Malignant lesions were more frequently observed in lower-extremity fractures compared with upper-extremity fractures. However, this difference did not reach statistical significance. The observed trend is consistent with the known predilection of primary malignant bone tumors for the lower extremity in pediatric patients [8, 10, 17]. Nevertheless, the limited number of malignant cases in this cohort likely reduced the statistical power to detect significant differences, and the results should therefore be interpreted with caution. Larger, multicenter studies may be required to assess the relationship more precisely between fracture location and lesion etiology.

As malignant lesions were overall uncommon, their potential consequences require a high level of diagnostic vigilance at initial presentation. In particular, pathological fractures occurring after absent or inadequate trauma, especially in the lower extremity or in older children, should not be presumed benign without careful assessment of the entire affected bone. Radiographic features such as ill-defined margins, cortical destruction, aggressive periosteal reaction, soft-tissue extension, or atypical fracture morphology should prompt advanced imaging and referral to a specialized musculoskeletal tumor center before definitive fixation is performed. At the same time, the treatment analysis suggests that the decision for surgery is not primarily determined by upper- versus lower-extremity location, as operative rates were comparable between groups. Instead, diaphyseal involvement emerged as the most relevant factor associated with operative management, likely reflecting the greater risk of instability, malalignment, and impaired mechanical integrity in shaft fractures. Therefore, treatment should be guided by fracture stability, lesion morphology, diagnostic certainty, and mechanical demand, while biopsy or lesion-directed procedures should be considered whenever the underlying pathology cannot be confidently classified as benign.

Taken together, particular diagnostic attention should be paid to children with lower-extremity fractures after absent, inadequate, or low-energy trauma, especially when the fracture appears disproportionate to the reported mechanism. In this cohort, these fractures were more often observed in female patients, associated with absent/non-adequate trauma, and showed a higher burden of systemic or multifocal bone conditions. Although malignancies were uncommon, their predominance in older children and lower-extremity fractures supports careful evaluation of the entire affected bone when clinical or radiographic red flags are present. Regarding treatment, operative rates were similar between groups, but treatment modalities differed significantly by location, and diaphyseal involvement was the main factor associated with surgery, suggesting that operative decision-making was primarily driven by mechanical instability.

### Limitations and strengths of the study

This study has several limitations. First, the retrospective design is associated with an inherent risk of selection bias and incomplete or inconsistent documentation. Second, the single-center setting may limit the external validity and generalizability of the findings. Third, the extended study period may have introduced heterogeneity in diagnostic workup and treatment strategies over the study period. Finally, although this study represents one of the larger pediatric cohorts of pathological fractures, the number of malignant lesions was small, limiting the statistical power for subgroup analyses.

Notwithstanding these limitations, the present study provides additional data on location-specific differences in pediatric pathological fractures. Recognition of such patterns may assist clinicians in considering underlying bone pathology, particularly in cases with atypical trauma mechanisms, and may support timely diagnostic evaluation and appropriate management.

## Conclusion

Pathological fractures of the upper and lower extremities in children demonstrate distinct clinical characteristics. Lower-extremity fractures were more frequently associated with absent or low-energy trauma and showed a trend toward a higher proportion of malignant underlying lesions, whereas upper-extremity fractures predominantly resulted from falls on level surfaces. These location-specific differences suggest that fracture location may provide valuable diagnostic information and should be considered during the evaluation of children presenting with pathological fractures.

## Data Availability

No datasets were generated or analysed during the current study.
